# Multi-omics studies reveal ameliorating effects of physical exercise on neurodegenerative diseases

**DOI:** 10.3389/fnagi.2022.1026688

**Published:** 2022-10-31

**Authors:** Yuhuai Guo, Shouli Wang, Xiaowen Chao, Ding Li, Ying Wang, Qihao Guo, Tianlu Chen

**Affiliations:** ^1^School of Kinesiology, Shanghai University of Sport, Shanghai, China; ^2^Center for Translational Medicine and Shanghai Key Laboratory of Diabetes Mellitus, Shanghai Jiao Tong University Affiliated Sixth People’s Hospital, Shanghai, China; ^3^Department of Gerontology, Shanghai Jiao Tong University Affiliated Sixth People’s Hospital, Shanghai, China

**Keywords:** physical activity, omics, Alzheimer’s disease, Parkinson’s disease, Huntington’s disease, amyotrophic lateral sclerosis

## Abstract

**Introduction:**

Neurodegenerative diseases such as Alzheimer’s disease, Parkinson’s disease, amyotrophic lateral sclerosis, and Huntington’s disease, are heavy burdens to global health and economic development worldwide. Mounting evidence suggests that exercise, a type of non-invasive intervention, has a positive impact on the life quality of elderly with neurodegenerative diseases. X-omics are powerful tools for mapping global biochemical changes in disease and treatment.

**Method:**

Three major databases were searched related to current studies in exercise intervention on neurodegenerative diseases using omics tools, including metabolomics, metagenomics, genomics, transcriptomics, and proteomics.

**Result:**

We summarized the omics features and potential mechanisms associated with exercise and neurodegenerative diseases in the current studies. Three main mechanisms by which exercise affects neurodegenerative diseases were summed up, including adult neurogenesis, brain-derived neurotrophic factor (BDNF) signaling, and short-chain fatty acids (SCFAs) metabolism.

**Conclusion:**

Overall, there is compelling evidence that exercise intervention is a feasible way of preventing the onset and alleviating the severity of neurodegenerative diseases. These studies highlight the importance of exercise as a complementary approach to the treatment and intervention of neurodegenerative diseases in addition to traditional treatments. More mechanisms on exercise interventions for neurodegenerative diseases, the specification of exercise prescriptions, and differentiated exercise programs should be explored so that they can actually be applied to the clinic.

## Introduction

Neurodegenerative diseases, including Alzheimer’s disease (AD), Parkinson’s disease (PD), Huntington’s disease (HD), amyotrophic lateral sclerosis (ALS), and the like, affect millions of people worldwide (Walker, 2007; Heemels, 2016). With the fast development of large-scale measurement platforms and omics data mining methods, various kinds of omics markers were identified and validated in animal and human studies. The early prediction and diagnosis of neurodegenerative diseases by blood omics markers or by the combination of omics markers and cognitive tests showed inspiring performances and are attracting more and more attention from clinicians (Jia et al., 2019; Palmqvist et al., 2021). Omics findings may also reflect the systematic changes of the whole body and thus can provide new insights to mechanism studies on disease onset and progression. For example, the close association between gut microbiota, microbial metabolites, and neurodegenerative disease progress and the interactions of gut, brain, and other organs shed a light on current neurodegenerative disease studies (MahmoudianDehkordi et al., 2019; Nho et al., 2019). At the pathway level, the pan-neurodegenerative signature was defined by an upregulation of pro-inflammatory and phagocytic pathways, and a downregulation of the mitochondrial oxidative phosphorylation (Noori et al., 2021). Neuroinflammation, together with a failure in neuronal energy metabolism and protein degradation is the substrates underlying the neurodegeneration (Noori et al., 2021).

Many studies showed that exercise is a good intervention for preventing the onset and alleviating the severity of neurodegenerative diseases with promising roles on various parts of the brain and body (Gubert et al., 2020). Gene expression profiles from physically active subjects correlated negatively with neurodegenerative diseases, AD, PD, frontal-temporal dementia (FTD), and HD (Santiago et al., 2022). A meta-analysis showed that regular physical activity (PA) performed by elderly people might play a certain protective role against AD (Santos-Lozano et al., 2016). Exercise can boost memory and dampen brain inflammation (De Miguel et al., 2021). The pathologic features and behavioral symptoms of AD caused by the accumulation of amyloid β-peptide in the hippocampus can be reversed by regular exercise (Hwang et al., 2022). Additionally, exercise could promote the motoneuron survival in ALS animals, protect against 1-Methyl-4-phenyl-1,2,3,6-tetrahydropyridine (MPTP)-induced neurotoxicity in the model mice of PD (Gerecke et al., 2010), and ameliorate striatal deficits in HD model mice (Pang et al., 2006). A study showed that in the high physical activity group, the significant expression genes modulation (WPSEG) was higher for Neurons, Dendritic Development, Synaptic transmission genes, and Axon Development (Sanfilippo et al., 2021). Recently, low-intensity exercise was reported to contribute to the stabilization of gut microbiota and the improvement of neuroplasticity and cognitive function in mice (Lai et al., 2021). Many large-scale studies have been launched focusing on the effects of the exercise intervention on the gut-brain axis and related diseases (Gubert et al., 2020; Royes, 2020).

Exercise intervention has many advantages. First, exercise is a non-invasive intervention, which will not cause additional pain and wounds to patients (Hwang et al., 2022). Compared with drugs and surgery, exercise intervention is more easily accepted by most patients. Second, exercise is a good lifestyle with broad benefits to physical and mental health (Paluska and Schwenk, 2000) and many disorders including age- or disease-related cognitive declines (Prakash et al., 2015; Chow et al., 2021), sarcopenia (Steffl et al., 2017), type 2 diabetes (Aune et al., 2015), rheumatoid arthritis (Katz et al., 2020), etc. The advantages of exercise intervention and the exciting omics findings on neurodegenerative diseases motivated us to review the omics studies on exercise and neurodegenerative diseases and to summarize the related features and potential mechanisms on disease management.

## Materials and methods

### Eligibility criteria

This review included studies that met the following criteria: (a) included studies on these four diseases: Alzheimer’s disease, Parkinson’s disease, Huntington’s disease, and amyotrophic lateral sclerosis, (b) articles using omics study, (c) the difference between groups was the presence or absence of physical activity or exercise intervention, (d) there were some specific markers rising or falling, (e) articles did not include reviews, conference papers, guidelines, dissertations, and non-English articles.

### Literature search

Two researchers (Guo and Chao) independently searched PubMed, ScienceDirect, and SpringerLink literature using the following keywords: (“Alzheimer” or “Parkinson” or “Amyotrophic lateral sclerosis” or “Huntington”) and (“physical activity” or “exercise”) and (“Metabolomics” or “Microbiomics” or “Genomics” or “proteomics” or “Transcriptomics”). Among these literatures, studies in the last decade or so were selected. If there was any inconsistency between the two investigators, a senior investigator (Wang) was invited to decide whether to include articles that met the inclusion and exclusion criteria and to approve the final list of articles.

### Data extraction

Literature information was extracted separately by two authors, while inconsistent information was judged by communication between the two authors. The data extracted from the final included literature included: omics method, sample type 1, sample type 2, sample size and grouping, markers and trends. The specific literature information included in this review is shown in Tables 1–4.

**Table 1:** Multi-omics studies of exercise and AD.

Omics	Sample type 1	Sample type 2	Sample size	Main changes of multi-omics after exercise
Genomics (Ngwa et al., 2021)	Human	blood	6-month program of aerobic exercise *n* = 8, controls *n* = 8	VPS52⬆
Transcriptomics (Hwang et al., 2022)	Mice	Brain tissue	nontransgenic control, Tg-PS2 WT, Tg-PS2m, Tg-PS2m/Ex	Cks2 ⬆, Cdc28 ⬆, cyclin D1 ⬇, caspase-3 ⬇
Protiomics (Rao et al., 2015)	Mice	Brain tissue	APP/PS1TG, WT	Aβ40 ⬇, Aβ42 ⬇
Proteomics (Teglas et al., 2020)	Mice	liver tissue	control (APP/PS1TG-C) *n* = 6, APP/ PS1TG-Ex *n* = 6, probiotic treated (APP/PS1TG-Pr) *n* = 6, combined (Ex and Pr, APP/ PS1TG-Ex-Pr) groups *n* = 6, WT *n* = 6	SOD2 protein ⬆
Proteomics (De Miguel et al., 2021)	Mice	blood	Run, control	clusterin ⬆
Metabolomics (Clark-Matott et al., 2015)	Mice	Brain tissue	21 WT (sed *n* = 12, Ex *n* = 9),19 mtDNA mutator mice (sed *n* = 10, Ex *n* = 9)	NAD+ ⬇, PARP-1 ⬆
Metabolomics (Gaitan et al., 2021) Genomics (Alvarez-Lopez et al., 2013; Allard et al., 2017)	Human(35, 85) Mice	blood (35, 85); Brain tissue (28);	23 asymptomatic late middle-aged adults with familial and genetic risk for AD (35); R1 sed, R1 run, P8 sed, and P8 run (*n* = 14/group) (28); 89 age > 55 volunteers (85)	BDNF*
Metabolomics (Wang et al., 2021; Li et al., 2022) Microbiomics (Wang et al., 2021) Genomics (Wierczeiko et al., 2021) Proteomics (Wang et al., 2021)	Mice	blood(29, 32, 78), Brain tissue (29, 32)	young control *n* = 7, young voluntary Ex *n* = 7 (32), saline, Aβ, Aβ + Run, Aβ + Run +40 Hz stim; (78); 5xFAD run, WT run, 5xFAD sed, WT sed (29)	GFAP*
Metabolomics (Lai et al., 2021)	Mice	feces	control group, Ex group, sur group, low intensity Ex + sur (Exe-l + Sur), Exe-m (middle intensity) + Sur, Exe-h (high intensity) + Sur	valeric acid ⬇
Microbiomics (Wang et al., 2020)	Mice	feces	control group *n* = 9, high-intensity interval training group *n* = 9	*Proteobacteria* ⬇, *Dorea* ⬆, *Dehalobacterium* ⬆, *Candidatus Arthromitus* ⬇
Microbiomics (Abraham et al., 2019)	Mice	Brain tissue, feces	APP/PS1TG-C *n* = 8, APP/PS1TG-Ex *n* = 8, APP/PS1TG-Pr *n* = 8, APP/PS1TG-Ex-Pr *n* = 8	*L. reuteri*⬆, butyrate ⬆
Microbiomics (Rosa et al., 2020)	Mice	feces	Sed, Ex, sed/Aβ1-40, Ex/Aβ1-40 (*n* = 10/group)	Firmicutes/Bacteroidetes⬆

Vacuolar Protein Sorting-Associated Protein 52 (VPS52); mice expressing the human presenilin-2 (Tg-PS2); wild type(WT); mice expressing the human presenilin-2 with the N141I mutation (Tg-PS2m); exercise (Ex); Cdc28 protein kinase regulatory subunit 2 (Cks2); APP/PSEN1 double-transgenic mice (APP/PS1TG); Amyloid-β (Aβ); superoxide dismutase (SOD2); sedentary (sed); Nicotinamide Adenine Dinucleotide (NAD); Poly ADP-ribose polymerase 1 (PARP-1); SAMR1 (R1); brain-derived neurotrophic factor (BDNF); glial fibrillary acidic protein (GFAP); surgery (sur). *Indicates that there is no consensus on the trend of the molecule.

**Table 2:** Multi-omics studies of exercise and PD.

Omics	Sample type 1	Sample type 2	Sample size	Main changes of multi-omics after exercise
Transcriptomics (Zhang et al., 2020)	Mice	Brain tissue	sham *n* = 5, PD *n* = 5, Ex *n* = 5	LOC102633466 ⬆，LOC102637865 ⬆，LOC102638670 ⬆
Transcriptomics (Klemann et al., 2018)	Mice	Brain tissue	saline *n* = 14, saline+Ex *n* = 14, MPTP *n* = 10, MPTP+Ex *n* = 13	L-DOPA ⬇, RICTOR ⬆
Transcriptomics (Viana et al., 2017)	Rat	Brain tissue	sed saline, sed MPTP, Ex saline, Ex MPTP (*n* = 11/group)	RAGE ⬆，DJ-1 ⬆
Proteomics (Ebanks et al., 2021)	Drosophila	mitochondria	Ex WT, non-exercised WT, Ex Pink1-, non-Ex Pink-1	(Tropomyosin-1 isoforms 33/34, Tropomyosin-2, Acyl-coenzyme A dehydrogenase, Isocitrate dehydrogenase, Enolase, Probable isocitrate dehydrogenase [NAD] subunit alpha, Glycerol-3-phosphate dehydrogenase [NAD (+)], Pyruvate dehydrogenase E1 component subunit beta, Aldo-keto reductase isoform C, Alcohol dehydrogenase, CG9992 isoform A) ⬇
Proteomics (Gerecke et al., 2012)	Mice	Brain tissue	WT SH + saline *n* = 8, WT SH + MPTP *n* = 10, BDNF+/− Ex saline *n* = 4, BDNF+/− Ex+MPTP *n* = 8, WT Ex+MPTP *n* = 6	BDNF*
Proteomics (Gerecke et al., 2010)	Mice	Brain tissue	SH saline *n* = 8, SH MPTP *n* = 10, 1 month Ex prior to MPTP *n* = 7, 2 months Ex MPTP *n* = 6, 3 months Ex MPTP *n* = 5	VDAC2 ⬇，Cofilin-1 ⬆，Profilin-1 ⬆，Echs1 ⬆，Apoa1 ⬆，Isovaleryl coenzyme A dehydrogenase ⬆，Dihydropteridine reductase ⬆，Protein-L-isoaspartate (D-aspartate) O-methyltransferase-1 ⬆，Argininosuccinate synthetase⬆，Hippocalcin ⬆，Chaperonin subunit 2 (beta) ⬇，Arl3-Gdp ⬆，BLBP ⬆
Proteomics (Dimatelis et al., 2013)	Rat	Brain tissue	SR, NSR, SNR, NSNR	(nucleoside diphosphate kinase B, enolase, triosephosphate isomerase, α-synuclein, tenascin-R, Ba1-667, brevican, neurocan core protein) ⬇
Metabolomics (Tan et al., 2021)	Human	feces	104 patients, 96 controls	SCFAs*, butyrate*
Metabolomics (Li et al., 2022)	Human	blood	Tai Chi *n* = 32, brisk walking *n* = 31, no-Ex *n* = 32	Fumaric acid ⬇, L-aspartic acid ⬇, pyroglutamic acidarginine ⬇, homocysteine ⬇，methionine sulfoxide ⬇, azelaic acid ⬆, L-fucose ⬆, adenosine ⬆, pipecolic acid ⬆
Microbiomics (O'Donovan et al., 2020)	Rat	feces	sed control *n* = 5, sed AAV-α-synuclein *n* = 5, Ex control *n* = 5, Ex AAV-α-synuclein *n* = 5	*Ruminococcaceae* ⬇, *Lachnospiraceae* ⬇

MPTP (1-methyl-4-phenyl-1,2,3,6-tetrahydropyridine); Levodopa (L-DOPA); rapamycin-insensitive companion of mTOR (RICTOR); receptor for advanced glycation end products (RAGE); DJ-1 gene (DJ-1); standard housing (SH) Voltage-dependent anion channel 2 (VDAC2); Enol coenzyme A hydratase, short chain 1 (Echs1); Apolipoprotein A-1 (Apoa1); ADP Ribosylation Factor Like GTPase 3 guanosine diphosphate (Arl3-Gdp); Brain lipid-binding protein (BLBP); stressed runners (SR); non-separated runners (NSR) stressed non-runners (SNR); non-separated non-runners (NSNR); short-chain fat acids (SCFAs). *Indicates that there is no consensus on the trend of the molecule.

**Table 3:** Multi-omics studies of exercise and HD.

Omics	Sample type 1	Sample type 2	Sample size	Main changes of multi-omics after exercise
Transcriptomics (Pang et al., 2006; Zajac et al., 2010)	Mice	Brain tissue	WT SH *n* = 11, HD SH *n* = 7, WT Ex *n* = 9, HD Ex *n* = 9(14); HD SH, WT SH, HD Ex, WT Ex	BDNF ⬆
Microbiomics (Gubert et al., 2022)	Mice	feces	SH *n* = 16, EE *n* = 16, Ex *n* = 16	Butyrate ⬇, valerate ⬇

Environmental enrichment (EE).

**Table 4:** Multi-omics studies of exercise and ALS.

Omics	Sample type 1	Sample type 2	Sample size	Main changes of multi-omics after exercise
Transcriptomics (Hashimoto et al., 2009)	mice	spinal cords	Ex, sed	C030039L03Rik ⬆, Ogn ⬆, Decr1 ⬆,Erdr1 ⬇, St14 ⬇, Morn1 ⬇,Bc17a ⬇, Tnni2 ⬇, Acta1 ⬇,Rbp3 ⬇, 201031E24Rik ⬇, Gria3 ⬇, Usp31 ⬇, Cnot3 ⬇, Hoxc8 ⬇, AI597479 ⬇, Tmem109 ⬇, Hspb7 ⬇, Fuk ⬇, Mospd3 ⬇, Pou2f1 ⬇, BC035947 ⬇, Rnu3b4 ⬇, Tuba8 ⬇, Gtf2f2 ⬇, Syp1 ⬇, Dock5 ⬇, St8sia1 ⬇, Mtss1 ⬇, Rtn1 ⬆, Lyz1 ⬇, Gmfg ⬇, Hck ⬇, Chcr1 ⬇, Atp2b1 ⬇, Chi313 ⬇, Lyz2 ⬇, Hist3h2ba ⬇, Nme4 ⬇, Dnmt3b ⬇, Tbx6 ⬇, S100a8 ⬇, Tuba8 ⬇
Transcriptomics (Ferraiuolo et al., 2009)	mice	lumbar spinal motoneurons	Ex *n* = 6, sed *n* = 6	Cntf ⬆, Lifr ⬆, CREB⬆, Narp ⬆, Acvr2a ⬆, NR1 E21 ⬇, Kcnd2 ⬇, Kcnd3 ⬆, Kcnk3 ⬆, Kcne2 ⬆, CamkIId ⬆, Gpr23 ⬇, Pctaire1 ⬇, Epas1 ⬆, Tie1 ⬆, Cxcr4 ⬆, Egln3 ⬆, Aurka ⬆, Hdgf ⬆, Tgfb1 ⬆, myomesin ⬆, EphA6 ⬆, Ptprm ⬆, Ctnna1 ⬆, Pcdh1, 12, 17, 18 ⬆, TrkB ⬆, Egr1 ⬆, aftiphilin ⬆, synaptosomal-associated protein 29 ⬆, Cacng8 ⬇, Cacng5 ⬇, calsequestrin ⬆, Dlg ⬆, chloride channel-2 ⬆
Transcriptomics (Julian et al., 2021)	human		460,376 subjects	C9ORF72 ⬇
Metabolomics (Desseille et al., 2017) Genomics (Desseille et al., 2017)	mice	blood, muscle tissue	Run ALS *n* = 18, Swim ALS *n* = 18, sed ALS *n* = 29, sed *n* = 20	glucose tolerance ⬆, circulating lactate ⬆ Glut4 mRNA ⬆, Gapdh mRNA ⬇, Pdk4 mRNA ⬇, LC3B-I protein ⬇, LC3B-II protein ⬇, Cd36 mRNA, Vldlr mRNA ⬆, Srebp-1c mRNA ⬆, Fas mRNA ⬆, Ucp3 mRNA ⬆, Dgat1 mRNA⬆, TAG ⬆

Voltage-dependent anion channel 2 (VDAC2); Enol coenzyme A hydratase, short chain 1 (Echs1); Brain lipid-binding protein (BLBP); brain-derived neurotrophic factor (BDNF); glyceraldehyde-3-phosphate dehydrogenase (GAPDH); Cluster of Differentiation 36 (CD36); Very Low Density Lipoprotein Receptor (VLDLR); Sterol Regulatory Element-Binding Protein 1c (Srebp-1c); Fatty Acid Synthase (Fas); Uncoupling protein 3 (Ucp3); diacylglycerol acyltransferase 1 (Dgat1); triacylglyceride (TAG); Motor neuron(MN); ciliary neurotrophic factor(CNTF); cAMP-response element binding protein(CREB); neuronal pentraxin 2(NARP); activin receptor II A (Acvr2a); Nova-2 favours E21 inclusion (NR1 E21); K^+^ voltage-gated channel, Shal- related family 2 (Kcnd2); voltage-gated K^+^ channels D3(Kcnd3); K^+^ channel, subfamily K, member 3 (Kcnk3);K^+^ voltage-gated channel, Isk-related subfamily, gene 2 (Kcne2); Ca^2+^/calmodulin-dependent kinase II (CamkIId); G protein-coupled receptor 23 (Gpr23); tyrosine kinase receptor 1 (Tie1); chemokine (C-X-C motif) receptor 4 (Cxcr4); egg laying nine homolog 3 (Egln3); Aurora kinase A (Aurka); hepatoma-derived growth factor (Hdgf); transforming growth factor-b1 (Tgfb1); Ephrin receptor A6(EphA6); protein tyrosine phosphatase l (Ptprm); catenin alpha (Ctnna1); protocadherins (Pcdh1, 12, 17, 18); early growth response 1 (Egr1); Ca^2+^ channel (Cacng8); Ca^2+^ channel (Cacng5); discs large homolog 5 (Dlg). *Indicates that there is no consensus on the trend of the molecule.

## Results

**Figure 1:**
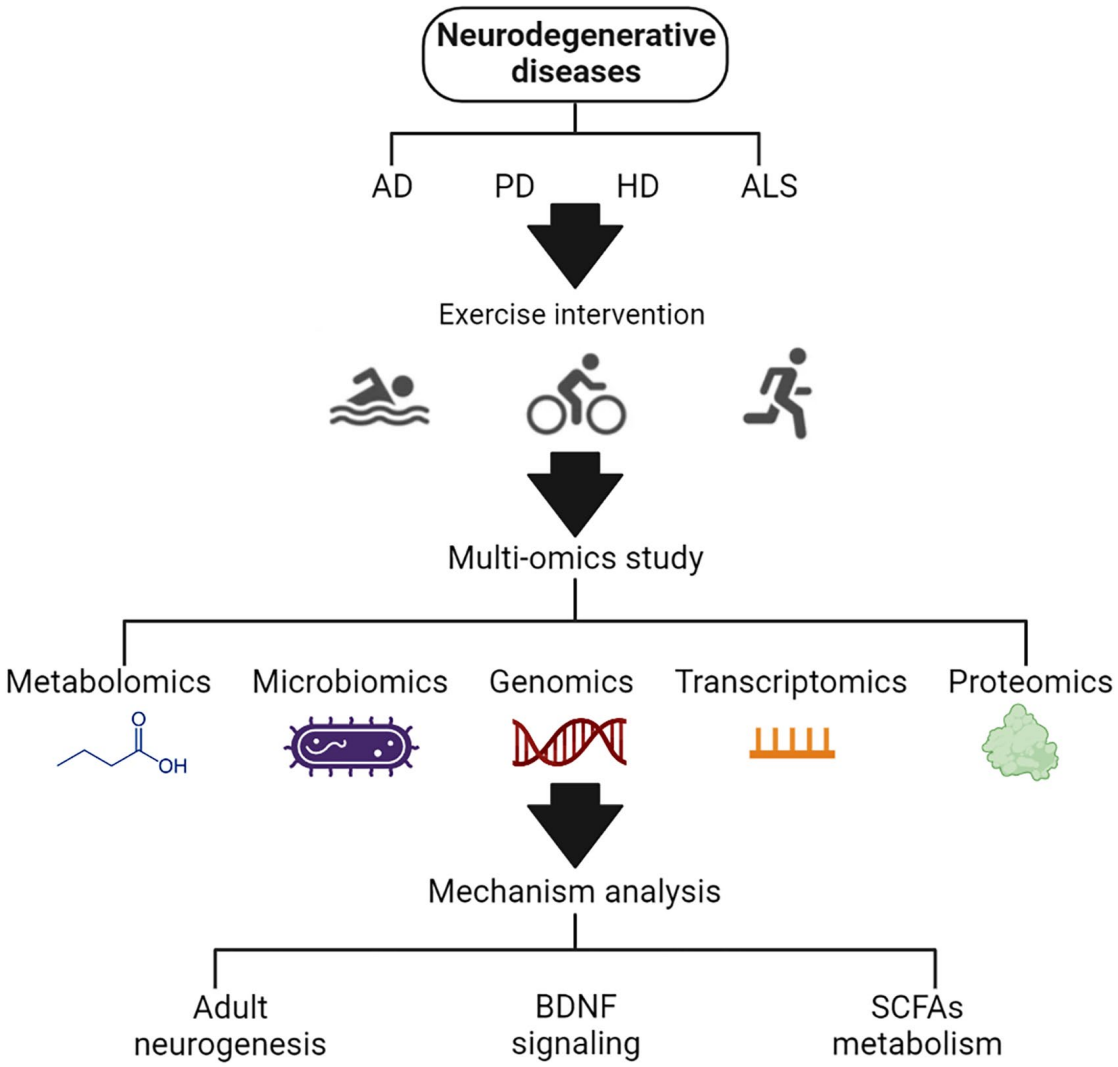
The schematic diagram of the effects of exercise on neurodegenerative diseases based on multi-omics analysis.

A total of 90 articles were found using various search strategies in three major databases. A total of five publications were excluded due to duplication or incomplete basic information. Another 11 papers were excluded because they were not relevant to our conditions of interest. When full text was examined, 6 because there were no omics studies, 3 because there were no clear markers and trends, 3 because of inconsistent outcome measures, and 5 because of non-target literature types. Finally, 57 articles were selected for analysis. Three main mechanisms by which exercise affects neurodegenerative diseases were summed up (Figure 1).

### Omics markers associated to exercise effects in AD patients

In the past decade, much has been learned about the conduct of clinical trials by which the efficacy of any proposed treatment for Alzheimer’s disease can be assessed (Masters and Beyreuther, 1998). Despite lots of research on AD, there still have no disease-modifying treatment (Lane et al., 2018). The earliest phase of AD (cellular phase) happens in parallel with accumulating amyloid β, inducing the spread of tau pathology (Scheltens et al., 2021). The risk of AD is 60–80% dependent on heritable factors, with more than 40 AD-associated genetic risk loci already identified, of which the APOE alleles have the strongest association with it (Scheltens et al., 2021). Accumulated clinical data support that the core cerebrospinal fluid (CSF) biomarkers, amyloid β (Aβ42), total Tau (T-Tau) and phosphorylated Tau (P-Tau), reflect key pathophysiological factors in AD (Blennow and Zetterberg, 2018). Biomarkers are of great significance for the diagnosis and prevention of AD and for observing the effect of exercise intervention. The potential biomarkers of exercise intervention for AD patients and animals are listed in Table 1.

The genomics analysis showed that exercise enhanced the AD model mice’s gene expression of BDNF (Alvarez-Lopez et al., 2013) and Glial fibrillary acidic protein (GFAP, participating in the formation and maintenance of cytoskeleton tension; Wierczeiko et al., 2021) in the hippocampus. It also increased the expression of vacuolar protein sorting 52 (VPS52) in MCI participants. VPS52 is a Golgi-associated protein involved in intracellular protein trafficking including amyloid precursor protein (Ngwa et al., 2021).

The transcriptomic analysis revealed that in AD mice the cell cycle regulatory gene, Cdc28 protein kinase regulatory subunit 2 (Cks2), was decreased while some cell cycle and apoptotic cell death-related factors, including cyclin D1, proliferating cell nuclear antigen, and cleaved caspase-3, were increased in the hippocampus of Tg-PS2m. The treadmill exercise reversed the altered expressions (Hwang et al., 2022).

The proteomics analysis showed that the levels of superoxide dismutase (SOD2) protein (Teglas et al., 2020), GFAP (Wang et al., 2021), and clusterin (De Miguel et al., 2021) were increased while Aβ40 (Rao et al., 2015) was decreased in AD mice after exercise. A lack of SOD2 protein might cause the liver to be under oxidative stress (Teglas et al., 2020). Intravenously injected clusterin binds to brain endothelial cells and can reduce neuroinflammatory gene expression in a mouse model of acute brain inflammation and Alzheimer’s disease (De Miguel et al., 2021).

The metabolomics analysis showed that exercise reduced the depletion of nicotinamide adenine dinucleotide (NAD+) and increased Poly [ADP-ribose] polymerase 1 (PARP-1) activity in AD model mice. The specific pathways altered in the brain were associated with an accelerated age-related accumulation of somatic mtDNA mutations (Clark-Matott et al., 2015). Exercise also regulated the metabolic changes of BDNF in AD patients (Gaitan et al., 2021), and decreased plasma valerate acid in AD model mice (Lai et al., 2021).

The microbiome study showed that exercise increased the abundance of *Lactobacillus reuteri*, a vitamin B12 producer, and some butyrate-producing bacteria in AD model mice (Abraham et al., 2019). At the phylum level, the relative abundance of Proteobacteria was significantly decreased by exercise. At the genera level, significantly increased *Dorea* and *Dehalobacterium*, and decreased *Candidatus Arthromitus* were observed in AD model mice (Wang et al., 2020). Meanwhile, the enriched pathways involved carbohydrate metabolism and signal transduction mechanisms, while the reduced pathways involved renal cell carcinoma, various types of N-glycan biosynthesis, glycan biosynthesis and metabolism, lipopolysaccharide biosynthesis, cell motility, and lipopolysaccharide biosynthesis (Wang et al., 2020). Sedentary Aβ1-40-exposed mice had reduced Firmicutes while increased Bacteroidetes abundance, so their Firmicutes/Bacteroidetes ratios decreased, and this was prevented by the practice of physical exercise (Rosa et al., 2020). The gut microbiota is considered an important factor in the progression of AD (Xin et al., 2019).

### Omics markers associated to exercise effects in PD patients

PD is the second most common neurodegenerative disorder (Reich and Savitt, 2019) and the most common serious movement disorder in the world, which affects about 1% of adults older than 60 years old (Samii et al., 2004). The term “parkinsonism” refers to a clinical syndrome, including bradykinesia, cogwheel rigidity, resting tremor, a slow shuffling gait, and imbalance (Reich and Savitt, 2019). These symptoms are due to the changes at different levels within the brain. The main pathological change is the progressive degeneration of neurons in the substantia nigra pars compacta, one of the nuclei constituting the basal ganglia (Lotankar et al., 2017). There are two subtypes of PD, tremor dominant (TD) and postural instability/gait difficulty (PIGD; Jankovic et al., 1990; Prakash et al., 2016). Symptoms of PD respond in varying degrees to drugs, and surgery offers hope for patients no longer adequately controlled in this manner (Samii et al., 2004). Exercise as adjunctive therapy may provide some positive effects for patients with PD (Gerecke et al., 2010). The potential biomarkers of exercise intervention for PD patients and animals are listed in Table 2.

The transcriptomics analysis showed that 3 IncRNAs, LOC102633466, LOC102637865, and LOC102638670 were significantly upregulated in the aerobic exercise trained PD mice. These molecules were mainly involved in the extracellular matrix receptor interaction, the Wnt pathway, and the PI3K/AKT/mTOR pathway (Zhang et al., 2020). Levodopa, a precursor of dopaminergic that has been used to treat PD motor symptoms and is considered the gold standard of therapy, was downregulated in the PD mice’s brain. Rapamycin-insensitive companion of mTOR (RICTOR) mediate the pathways in the ventral tegmental areas and dorsolateral striatum, which was involved in energy metabolism and cellular stress. RICTOR was upregulated in PD mice with physical exercise (Klemann et al., 2018).

The proteomics analysis showed that exercise downregulated tropomyosin-1 isoforms 33/34, tropomyosin-2, acyl-coenzyme A dehydrogenase, isocitrate dehydrogenase, enolase, probable isocitrate dehydrogenase [NAD] subunit alpha, glycerol-3-phosphate dehydrogenase [NAD (+)], pyruvate dehydrogenase E1 component subunit beta, aldo-keto reductase isoform C, alcohol dehydrogenase, and CG9992 isoform A in PD drosophila. These molecules might be candidates to develop therapeutic approaches in PD (Ebanks et al., 2021). Exercise can reverse many of the PD-related changes by downregulating the levels of hippocampal proteins functionally associated with energy metabolism (nucleoside diphosphate kinase B, enolase, and triosephosphate isomerase) and synaptic plasticity (α-synuclein, tenascin-R, Ba1-667, brevican and neurocan core protein) in the non-lesioned hemisphere of PD model rats (Dimatelis et al., 2013). Animal studies also showed that voltage-dependent anion channel 2 (VDAC2; located on the outer membrane of mitochondria and functions to regulate much of the activity of these organelles) and Chaperonin subunit 2 (beta) were decreased, while Cofilin-1, Profilin-1 (cytoskeletal proteins and assembly regulators), Echs1 (energy metabolism), Apoa1, Isovaleryl coenzyme A dehydrogenase, Protein-L-isoaspartate (D-aspartate) O-methyltransferase-1, Argininosuccinate synthetase (amino acid transport and metabolism), Hippocalcin (cytoplasmic signaling molecules), Arl3-Gdp, and Brain lipid-binding protein (BLBP) were increased in substantia nigra and striatum of PD mice after exercise (Gerecke et al., 2010).

The metabolomics analysis showed that the early-stage PD patients after Tai Chi training, fumaric acid, L-aspartic acid, pyroglutamic, acid-arginine, homocysteine, and methionine sulfoxide were downregulated, while azelaic acid, L-fucose, adenosine, and pipecolic acid were upregulated, which showed the beneficial effects of exercise on the motor symptoms including gait and balance (Li et al., 2022). Low levels of PA were related to low levels of SCFAs in PD patients (Tan et al., 2021).

The microbiome analysis showed that in the Firmicute phylum, Lachnospiraceae and Ruminococcaceae families were significantly decreased in the feces of the PD model rats compared to their sedentary counterparts. Both of them were associated with gut health (O'Donovan et al., 2020).

### Omics markers associated to exercise effects in HD patients

HD is expressed ubiquitously throughout the body, affecting both the brain and periphery (Li et al., 1993), including along the gastrointestinal (GI) tract (Moffitt et al., 2009). GI abnormalities were previously demonstrated in the juvenile-onset R6/2 HD mouse model, with impairment of gut motility and malabsorption of food inversely correlated with body weight (van der Burg et al., 2011). Gut permeability and decreased colon length were also seen in R6/2 HD mice (Stan et al., 2020). Evidence of GI microbial population imbalance (gut dysbiosis) was found in R6/1 HD mice by profiling the gut microbiome (Kong et al., 2020). These preclinical findings were followed by the gut microbiome characterization in gene-positive HD subjects (Du et al., 2020; Wasser et al., 2020). These not only showed the first clinical evidence of gut dysbiosis in HD but also demonstrated associations with cognitive performance and clinical outcomes (Wasser et al., 2020).

The potential biomarkers of exercise intervention for HD animals are shown in Table 3. Short-chain fatty acids (SCFAs) and branched-chain fatty acids (BCFAs) are both microbiota-derived metabolites produced by the fermentation of dietary fiber and amino acids, respectively (Gubert et al., 2022). A report that investigated SCFAs and BCFAs fecal concentration showed that the fecal SCFAs butyrate and valerate concentrations of mice increased after voluntary wheel running (Rosa et al., 2020). Disruption of BDNF gene expression is a key to the development of symptoms in HD (Zajac et al., 2010). A recent study using the R6/1 HD mice model showed that the BDNF mRNA levels of striatal in the HD standard housed (SH) mice were 60% lower than wild-type (WT) SH mice (Zajac et al., 2010). Running partially rescued BDNF’s expression in the HD runner’s group, and the level was significantly higher than that of HD SH group (*p* < 0.05). Another report showed that the deficit in total BDNF gene expression in the female HD hippocampus was ameliorated by running (Pang et al., 2006).

### Omics markers associated to exercise effects in ALS patients

Amyotrophic lateral sclerosis (ALS) is an idiopathic, fatal neurodegenerative disease of the human motor system (Kiernan et al., 2011). It is characterized by the degeneration of both upper and lower motor neurons, leading to muscle weakness and eventual paralysis (Brown and Al-Chalabi, 2017). It begins with small symptoms such as muscle twitches or weakness. Eventually, ALS robs a person of all muscle control, resulting in paralysis, the inability to speak, and inevitably, death (Owens, 2017). The initial presentation of ALS can vary between patients; some present with spinal-onset disease (that is, the onset of muscle weakness of the limbs), but other patients can present with a bulbar-onset disease, which is characterized by dysarthria (difficulty with speech) and dysphagia (difficulty swallowing; Hardiman et al., 2017). The potential biomarkers of exercise intervention for ALS patients and animals are listed in Table 4.

In a transcriptomics study, aerobic exercise enhanced *Glut4*, *Cd36*, *Dgat1,* and *Vldlr* expression, and decreased the *Gapdh* level in muscles of ALS mice (Desseille et al., 2017). Interestingly, the expression profile of *Fas* and *Ucp3* paralleled those of *Srebp-1c*, with a decrease in muscles of sedentary ALS mice, and a significant increase in aerobic exercise-trained muscles of ALS mice (Desseille et al., 2017). The aerobic exercise shift to an anaerobic glycolytic pathway was associated with enhanced fat storage in the muscles of ALS mice. It is likely resulting from: 1. lipid uptake, as suggested by the increase of *Vldlr* and *Cd36* expression levels; 2. triacylglycerols synthesis, as suggested by the aerobic exercise-induced expression of lipogenesis genes such as *Fas*, *Acc1*, and *Dgat1* (Schenk and Horowitz, 2007).

A study using microarrays demonstrated that aerobic exercise resulted in specific gene expression changes in mouse spinal cords. The *Gria3* is a glutamate receptor called GluR3, whose antisense peptide nucleic acid-targeting GluR3 delayed disease onset and progression in the SOD1 G93A mouse model of familial ALS (Rembach et al., 2004). The elimination of *St8sial* coding GD3 synthase improved memory and reduced amyloid-beta plaque load in AD model mice (Bernardo et al., 2009). Reticulon family members including *Rtn1* coding reticulon1 modulated BACE1 activity and amyloid-beta peptide generation (He et al., 2004). Another report for humans showed that 52% of clinically validated ALS-related genes are differentially expressed following acute exercise. This enrichment is statistically significant, including down-regulation of *C9ORF72*. G4C2-repeat expansion of C9ORF72 is the most common genetic risk factor for ALS (Julian et al., 2021).

In a metabolomics study, aerobic exercise training in ALS mice completely restored glucose tolerance and induced a significant increase in the levels of circulating lactate and triacylglycerols. This exercise also decreased LC3B-I and LC3B-II proteins compared with sedentary controls (Desseille et al., 2017). ALS-induced glucose intolerance, albeit well established in human ALS patients (Julian et al., 2021). Accordingly, blood lactate levels were significantly increased by the aerobic exercise, strongly suggesting that the produced pyruvate is used, a least in part, to enhance the anaerobic glycolytic pathway in muscles of ALS mice (Desseille et al., 2017).

## Mechanistic roles of omics features in exercise intervention and neurodegenerative diseases

### Adult neurogenesis

Differing from prenatal neurogenesis, adult neurogenesis is a process in which neurons are generated from neural stem cells in the adult. In most mammals, new neurons are born throughout adulthood in two regions of the brain (Ernst and Frisen, 2015). The first region is the subgranular zone (SGZ), part of the dentate gyrus of the hippocampus (Dayer et al., 2003), where neural stem cells give birth to granule cells (implicated in memory formation and learning). The second region is the subventricular zone of lateral ventricles (SVZ), which can be divided into three microdomains, lateral, dorsal, and medial (Fiorelli et al., 2015). Neural stem cells migrate to the olfactory bulb through the rostral migratory stream where they differentiate into interneurons and participate in the sense of smell (Ernst et al., 2014).

In humans, however, few olfactory bulb neurons are generated after birth (Ernst et al., 2014). Adult neurogenesis is an essential brain mechanism involving in proliferation and differentiation of neural progenitor cells and synaptic plasticity of neurons (Sun, 2018). This unique form of neural development has attracted much interest not only as a model system to investigate brain development, but also to understand hippocampal functions. Because newborn granule neurons have roles in several cognitive functions, including spatial learning and retention, memory retrieval, forgetting, and clearance of memory traces (Deng et al., 2010).

Adult neurogenesis regulates multiple aspects related to mood and memory (Sun, 2018). Aberrant adult neurogenesis is closely linked to intellectual disabilities, neurodegenerative and neuropsychiatric disorders (Weissleder et al., 2019). For young APP/PS1 (AD model) mice that develop human AD-like amyloid pathology, exercise elevated their key proteins involved in synaptic plasticity, such as BDNF and synaptophysin (Wang et al., 2021). Exercise could alter gut microbiota profiles that might potentially affect tau pathology, neuroinflammation, and synaptophysin protein expression (Wang et al., 2021).

Additionally, exercise could enhance several metabolic pathways (e.g., glycine, serine, and threonine metabolism, phenylalanine, tyrosine, and tryptophan biosynthesis, the aminoacyl-tRNA biosynthesis), and accordingly affect key metabolites (e.g., L-valine, glucosamine, formyl anthranilic acid, and myristic acid), which might directly or indirectly affect the synaptic plasticity (Wang et al., 2021). Some studies suggested that multifactor intervention-mediated metabolites participated in the pathways of synaptic plasticity and neuronal function (Li et al., 2022). Left carotid artery exposure surgery reduced the expression of two synaptic proteins, postsynaptic density protein 95 (PSD-95) and synapsin-1, which could be attenuated by exercise (Lai et al., 2021). Synucleinopathies such as PD and Lewy bodies dementia is caused by over-expression of alpha-synuclein produces insoluble inclusion bodies (Ryskalin et al., 2018). Maternal separation upregulated hippocampal proteins including α-synuclein in the non-lesioned hemisphere, and this upregulation could be attenuated by exercise (Dimatelis et al., 2013). Exercise attenuated the Left carotid artery exposure surgery effect, which reduced brain cell genesis including glial fibrillary acidic protein (GFAP)-positive cells (Lai et al., 2021). Li et al. found that 4 weeks of multifactor intervention can significantly increase the production of newborn cells (BrdU^+^ cells) and immature neurons (DCX^+^ cells) in the hippocampus and lateral ventricle of Aβ oligomer-induced mice (Li et al., 2022). What’s important is that the multifactor intervention could promote differentiating BrdU^+^ cells into neurons (BrdU^+^ DCX^+^ cells or BrdU^+^ NeuN^+^ cells) and astrocytes (BrdU^+^ GFAP^+^ cells) in the hippocampus (Li et al., 2022). Besides, exercise reduced tau phosphorylation *via* inhibiting p-GSK3β activity in young APP/PS1 mice (Wang et al., 2021). In healthy mice, exercise attenuates neuroinflammation and glial cell line-derived neurotrophic factor (GDNF) reduction induced by left carotid artery exposure surgery. This effect is beneficial for maintaining astrocytes in an A2 phenotypic so that exercise can block surgery-induced dendritic arborization impairment (Lai et al., 2021).

**Figure 2:**
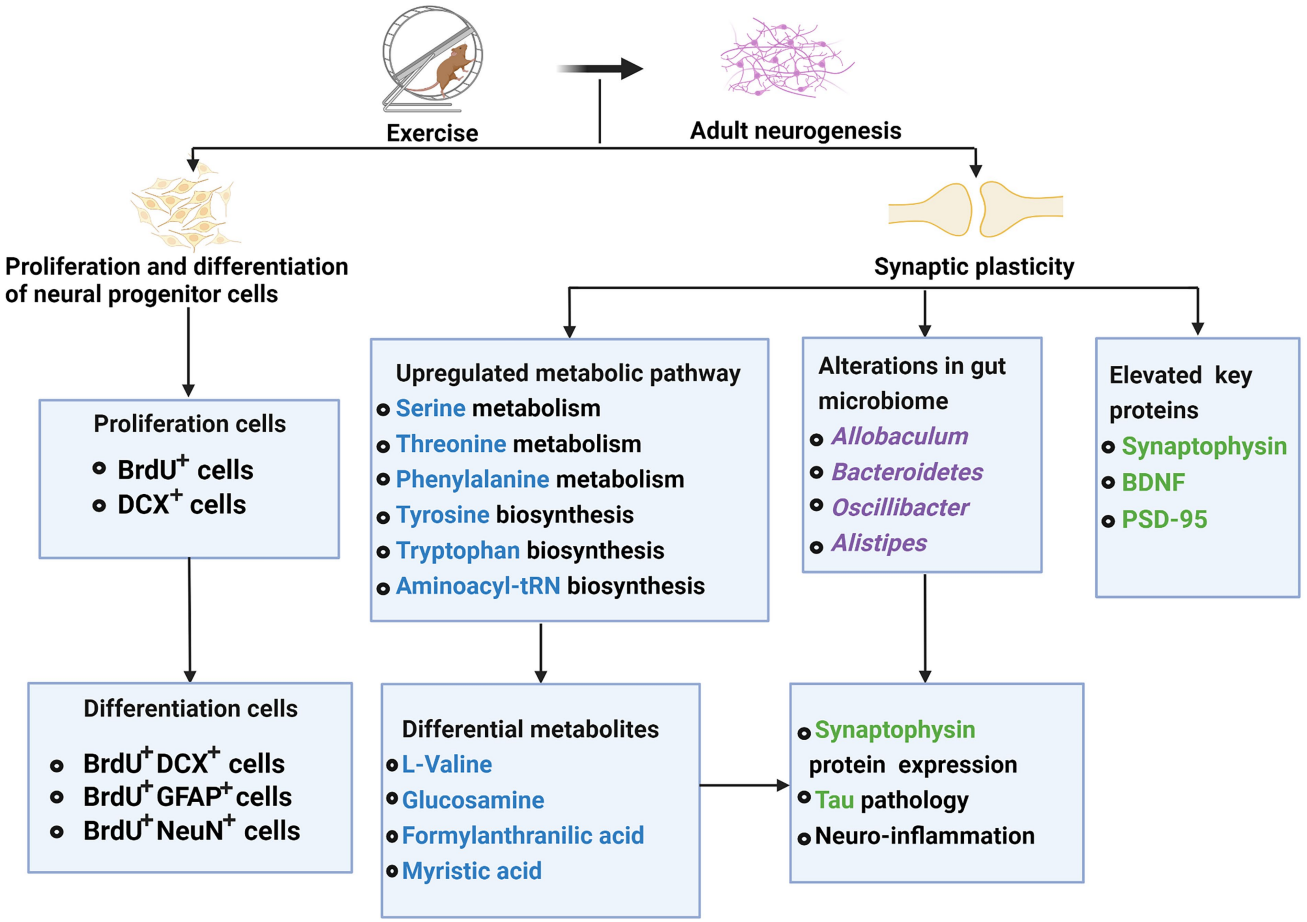
Effects of exercise on adults’ neurogenesis in neurodegenerative diseases.

Taken together, exercise interventions exert a facilitative effect on adult neurogenesis and cognitive function by improving neuronal synaptic plasticity, protecting the generation of new cells, and promoting the proliferation and differentiation of neural progenitor cells (Figure 2).

### Brain-derived neurotrophic factor signaling

Brain-derived neurotrophic factor (BDNF) is a key molecule involved in plastic changes related to learning and memory (Miranda et al., 2019). Changes in BDNF expression are associated with both normal and pathological aging and also psychiatric disease, in particular in structures important for memory processes such as the hippocampus and parahippocampal areas (Miranda et al., 2019). BDNF is expressed in the brain. The highest levels of BDNF’s mRNAs are found within the hippocampus, where these mRNAs are expressed in the overlapping but distinct distribution in neurons (Timmusk et al., 1993).

Reduced expression of BDNF has a crucial role in the pathogenesis of AD (Figure 2). It is characterized by the formation of neuritic plaques consisting of amyloid-beta (Aβ) and neurofibrillary tangles composed of hyperphosphorylated tau protein (Fletcher et al., 2018). A growing body of evidence indicated a potential protective effect of BDNF against Aβ-induced neurotoxicity in AD mouse models (Fletcher et al., 2018). BDNF and its receptor tropomyosin receptor kinase B (TrkB) together can induce a dendritic growth (Cheung et al., 2007).

Exercise can prevent the specific down-regulation of BDNF in patients with neurodegenerative diseases. According to Kimberly M. Gerecke’s research (Gerecke et al., 2012), the stereological estimates of substantia nigra pars compacta (SNpc) dopaminergic (DA) neurons were protected against the neurotoxicity induced by 1methyl-4-phenyl-1,2,3,6-tetrahydropyridine (MPTP) in wild type (WT) mice received 90 days of exercise *via* unrestricted running. However, heterozygous for the BDNF gene (BDNF+/2) mice that received 90 days of unrestricted exercise were not protected from MPTP-induced SNpc DA neuron loss (Gerecke et al., 2012). Proteomic analysis compared substantia nigra (SN) and striatum from 90-days exercised WT and BDNF+/ 2 mice showed differential expression of proteins related to energy regulation, intracellular signaling, and trafficking (Gerecke et al., 2012). These results suggested that a full genetic complement of BDNF is critical for the exercise-induced neuroprotection of SNpc DA neurons (Gerecke et al., 2012).

Long-term exercise can enhance hippocampal BDNF gene expression in senescent female mice. The senescence-accelerated mouse P8 (SAMP8) is considered a useful non-transgenic model for studying progressive cognitive decline and AD. Before long-term exercise intervention in old SAMP8 mice, expressions of BDNF and its receptor TrkB was examined first. Researchers found that both genes were under-expressed in sedentary SAMP8 (P8sed) compared with sedentary senescence-resistant mice (control group). After 6 months of voluntary wheel running in 10-month-old female SAMP8 mice, the phenotypic features associated with premature aging (i.e., skin color and body tremor) were improved, and the vascularization and BDNF gene expression were enhanced in the hippocampus compared with controls (Alvarez-Lopez et al., 2013). Transcriptomic studies revealed that upregulation of BDNF Exon 3 transcript was the only common effect of running in both female and male mice, which might be the main reason for the downstream beneficial effects of running on the HD disease progression (Zajac et al., 2010).

**Figure 3:**
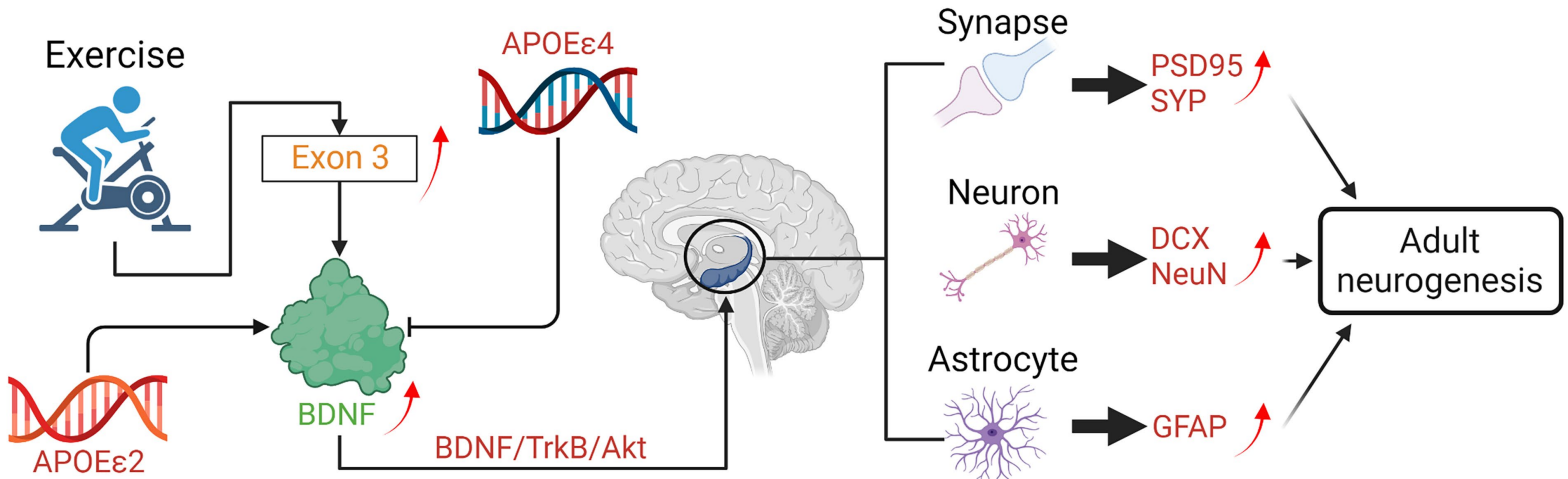
Effects of exercise on the neurogenesis mediated by BDNF in neurodegenerative diseases.

Some findings suggested that APOE genotype is an essential factor for exercise-induced BDNF upregulation in elderly mild cognitively impaired subjects (Allard et al., 2017). Thus, the ε4 allele of the APOE gene might influence the neuroprotective benefit induced by PA (Allard et al., 2017). Jaisalmer et al. used the power in the alpha band in 113 healthy subjects to measure the effect of exercise on the degree of brain aging and explored the effect of ε4 allele carrier status on exercise-induced neuroprotection (de Frutos-Lucas et al., 2020). Conversely, the presence of the ε2 allele seems to correlate with a protective phenotype in neurodegenerative disorders (Serrano-Pozo et al., 2015). There is study showed that the overall effect of the ε4 allele alone seems non-significant (Mui et al., 1995), while the possible protective role of the ε2 allele form was poorly explored (Buée et al., 1996). Diego Albani et al. highlighted an association between ALS and the ε2 allele, which was generally considered neuroprotective (Albani et al., 2017). Their result showed that among the group of PA the ε2 allele was significantly more frequent in ALS patients than in physically inactive patients (Albani et al., 2017). The ε4 allele of *APOE* has been associated with the development of the atherosclerosis (Altenburg et al., 2007) and cardiovascular disease (Lopez et al., 2014), both of which increase AD risk. The ε3 and ε2 alleles of *APOE* confer neutral and protective risk, respectively (Wang et al., 2019; Figure 3).

Besides BDNF, the Glial cell-derived neurotrophic factor (GDNF), and the neurotrophin nerve growth factor (NGF) are also important neurotrophic factors beneficial for the survival, maintenance, and regeneration of specific neuronal populations in the adult brain. Depletion of these neurotrophic factors has been linked with disease pathology and symptoms, and replacement strategies are considered potential therapeutics for neurodegenerative diseases (Allen et al., 2013). Growth factors like NGF are important in the neuronal plasticity and survival of forebrain cholinergic neurons (cerebral cortex, hippocampus, basal forebrain, and hypothalamus), which are memory-related (Lärkfors et al., 1987). GDNF is an important growth factor for the development, survival, and maintenance of midbrain dopaminergic neurons (Deister and Schmidt, 2006). GDNF can protect nigrostriatal dopamine neurons against the effects of 6-hydroxydopamine in the aged as well as young adult rats (Fox et al., 2001). As for the relationships between neurotrophic factors, in an experiment with 9 active men, the mRNA relative expression of NGF and GDNF mRNA increased after exercise (Peake et al., 2017). Exercise can increase the striatal level of GDNF in the Parkinson’s disease (PD) mice model which will attenuate L-3,4-dihydroxyphenylalanine (L-DOPA) -induced dyskinesia (Alves et al., 2019; Speck et al., 2019). Exercise-induced enhancement of NGF within the septo-hippocampal pathway represents a key avenue for aiding failing septo-hippocampal functioning and therefore has significant potential for the recovery of memory and cognition in several neurological disorders (Hall et al., 2018). All these results above suggest that exercise-induced upregulation of NGF and GDNF contributes to the attenuation of neurodegenerative diseases.

### Short chain fatty acids metabolism

SCFAs are saturated fatty acids with a chain length ranging from one to six carbon atoms. They are the main products of the fermentation of dietary fiber modulated by gut microbiota (Silva et al., 2020) and can cross the blood–brain barrier (BBB; Mitchell et al., 2011). So, they are important molecules connecting the brain and gut. Among the SCFAs, acetate, propionate, and butyrate are most abundant in the human body, while other SCFAs, such as formate, valerate, and caproate, are produced in lesser amounts (Cummings et al., 1987). SCFAs are considered key candidate mediators in the microbiota-gut-brain communication (Silva et al., 2020).

Greater α-diversity and enriched butyrate were reported in athletes compared to sedentary controls (Cheatham et al., 2022). Meanwhile, exercise increased the levels of butyrate and the butyrate-producing bacteria, such as *Roseburia hominis*, *Faecalibacterium pausnitzii*, and Ruminococcaceae, in both rodents and humans (Gubert et al., 2020). Butyrate is a well-known SCFA with an anti-inflammatory effect. They can inhibit cytokine release, increase the expression of tight-junction proteins, and consequently decrease the diffusion of lipopolysaccharide into the circulation (Abraham et al., 2019). It can activate intracellular signaling pathways to regulate the immune and inflammatory responses *via* working on their receptors or target proteins, such as nuclear factor κB (Lai et al., 2021). Butyrate was also reported to stimulate neural proliferation in the dentate gyrus in mice and has been used to induce neurogenesis after ischemic brain insult in adult rodents (Kim et al., 2009; Lai et al., 2021). Interestingly, after exercise, the abundance of Eubacteria, Roseburia, and Clostridia in APP/PS1TG mice were higher than that in the APP/PS1TG-Pr (probiotic received) mice, indicating that compared to probiotic treatment, exercise can produce more butyrate producer strains (Ngwa et al., 2021). Further on, APP/PS1TG-C and APP/SP1TG-Pr mice had significantly lower levels of *B. proteoclasticus* than WT, APP/SP1TG-Ex (exercise), and APP/SP1TG-Ex-Pr groups, suggesting that exercise and exercise combined with probiotic treatment may have beneficial to AD patients (Abraham et al., 2019). Besides AD, exercise-induced alteration of butyrate level was negatively associated with PD progression in human and animal studies (Rane et al., 2012; Tan et al., 2021).

On the contrary, valeric acid was reported to increase inflammatory responses and impair brain cell genesis (Lai et al., 2021). Lai’s surgery and exercise research showed that although exercise increased the blood concentrations of most SCFAs in young adult mice after surgery, valeric acid was the only decreased one (Lai et al., 2021). The mice receiving the feces from the exercise mice had better learning and memory than those receiving the feces from the control mice.

**Figure 4:**
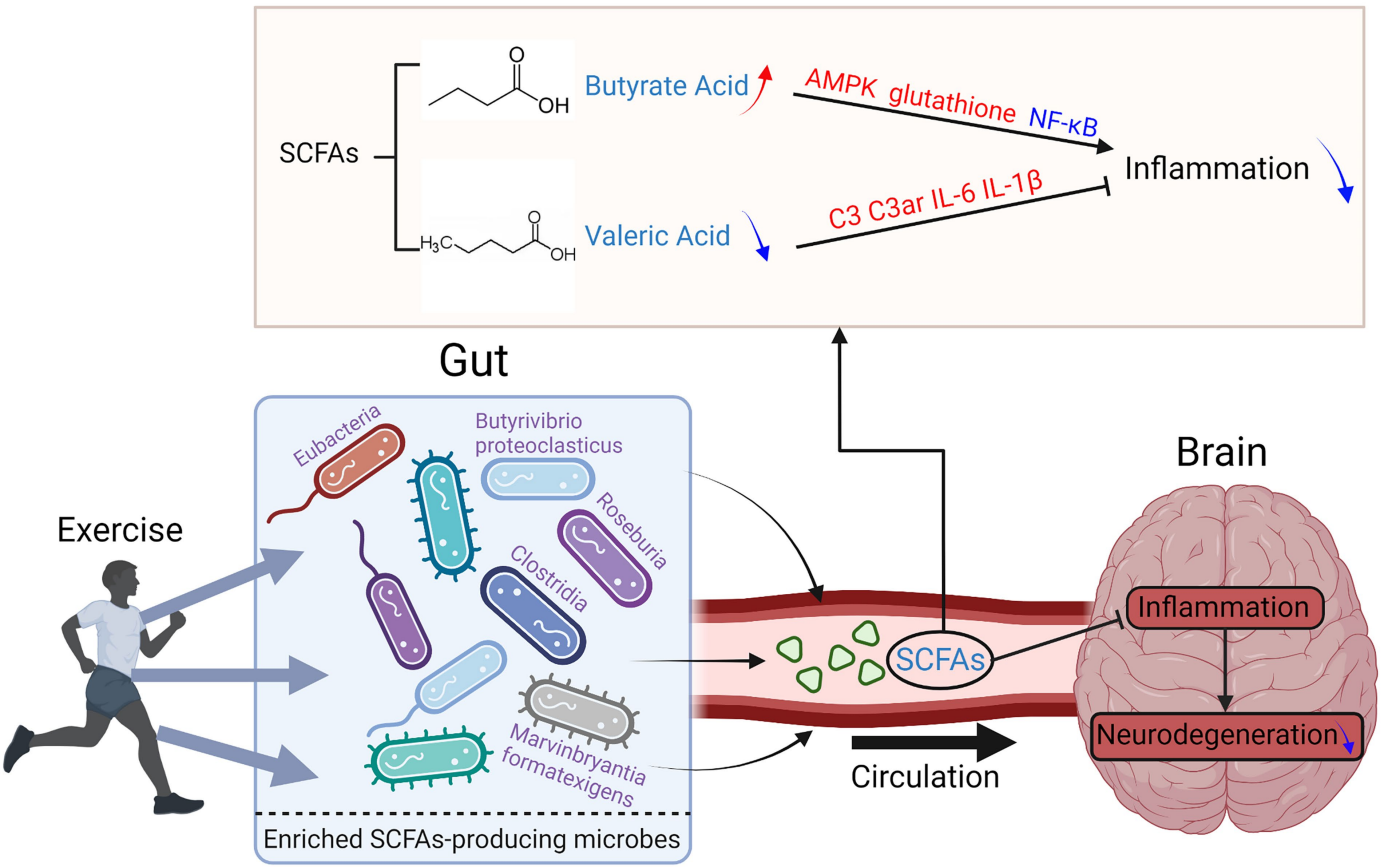
Exercise reduces the inflammation levels of the brain in neurodegenerative diseases by regulating the composition of SCFAs-producing gut bacteria.

In conclusion, exercise is effective in improving neurodegenerative diseases by regulating the levels of SCFAs producing bacteria and the levels of SCFAs. A diet with high fiber would result in an enrichment of fiber fermentation and SCFA producers, and was shown to benefit cognitive performance in both human and animal models (Hanstock et al., 2004). Exercise could improve the intestinal microbiota of patients with neurodegenerative diseases, thereby regulating the production of SCFAs and further promoting the regulation of the integrity of the intestinal epithelial barrier and blood–brain barrier, neuronal survival, and reducing the inflammatory response (Abraham et al., 2019; Lai et al., 2021; Tan et al., 2021; Figure 4). Therefore, the combination of exercise and diet may be an effective and feasible way of neurodegenerative disease control.

There are other pathways of inflammatory suppression that can ameliorate neurodegenerative diseases under exercise intervention, for example, researchers analyzed plasma proteins from mice after running a long distance and isolated a protein clusterin, with anti-inflammatory effects, which is significantly higher in the blood of long-distance running mice than in never exercising mice (De Miguel et al., 2021). Receptors on the endothelial cells of cerebral blood vessels can bind clusterin, thereby transmitting chemical signals from the blood to the brain. Experiments have shown that depletion of serum clusterin, the blood from long-distance running mice largely loses its effect on reducing neuroinflammation in sedentary mice. In contrast, intravenous administration of clusterin reduced brain inflammation in both acute brain inflammation mice and AD mice models (De Miguel et al., 2021). Also, researchers found that clusterin is an astrocyte-derived synaptogenic and anti-amyloid factor. Overall, the combination of these activities mentioned above may influence the progression of the late-onset AD (Chen et al., 2021). Tumor necrosis factor-α (TNF-α) is a cytokine involved in systemic inflammation. Dysregulation of TNF-α production has been implicated in AD (Swardfager et al., 2010). Interleukin-1β (IL-1β), a master regulator of neuroinflammation produced by activated inflammatory cells of the myeloid lineage, in particular microglia, plays a key role in the pathogenesis of acute and chronic diseases of the peripheral nervous system and CNS (Facci et al., 2018). Many studies show that significant anti-inflammatory effects are achieved by inhibiting of TNF-α and stimulating IL-1Ra, thereby limiting the IL-1β signaling (Karstoft and Pedersen, 2016; Pedersen, 2017). Besides, older adults with cognitive impairment after exercising showed a significant decrease in TNF-α compared to control groups, with high heterogeneity (Hildreth et al., 2015). Also, another study showed a significant decrease in serum IL-1β in male individuals after aerobic training compared to the placebo group (Baker et al., 2010). All of these results indicate that neuroinflammation plays a significant role in the development of neurodegenerative diseases, and exercise-induced anti-inflammatory effects may improve neurodegenerative diseases, with promising therapeutic value in neurodegenerative diseases.

## Discussion

Exercise intervention is a feasible and effective way of preventing the onset and alleviating the severity of neurodegenerative diseases. Omics tools demonstrated their impressive power for complex systematic disease studies. Currently, more and more omics studies are focusing on the relationship between exercise and neurodegenerative diseases. We have summarized recently identified features and their mechanistic roles associated to exercise effects on neurodegenerative diseases. Besides adult neurogenesis, BNDF, and SCFA metabolism, there are some other associated pathways like Firmicutes/Bacteroidetes ratio, ApoE expression, neurofilament light chain (NfL), GFAP et al. These mechanisms are also worthy of study.

Most of the studies mentioned above have demonstrated that exercise is beneficial to disease improvement. However, among numerous types of exercises, aerobic exercises like Tai Chi, yoga, swimming, and jogging seem to be better ways to improve the cognition and execution of patients with neurodegenerative diseases. Additionally, exercise should be done at a moderate intensity for at least 30 min. Higher exercise intensity seemed to have the opposite effect on improvement while shorter durations might have no significant effect. Different types of exercise attenuate the neurodegenerative disease through different pathways (Curtis et al., 2022). Aerobic and anaerobic training are both important for the release of myokines to improve systemic homeostasis and decrease inflammation. Aerobic endurance exercise has historically been shown to have the ability to increase nuclear and mitochondrial gene expression. Endurance training increased autophagic and mitophagic flux to turnover mitochondria. Resistance training, when performed after endurance training, amplified the induction of PGC-1α and PPAR-β/δ and led to an increased amount of mitochondrial biogenesis. High-intensity interval training modulates the autonomic nervous system and heart rate by decreasing the activity of the parasympathetic system and increasing the activity of the sympathetic system. All of these changes could benefit patients with neurodegenerative diseases (Curtis et al., 2022). Unfortunately, most of the current omics studies are steady-state aerobic exercises. Other types of exercise are rarely examined. Exercise intensity, time, and resistance methods are not fully considered. Some common training methods such as resistance training, HIIT, and LSD are not used in the interventions. More comprehensive and in-depth studies are expected. As far as we know, several well-designed large-scale studies are ongoing and more findings will be published in the near future.

There are also differences and commonalities between the effects of exercise interventions on different neurodegenerative diseases. The following studies illustrate the potential mechanisms by which exercise improves Alzheimer’s disease (AD) from different perspectives. Two studies have confirmed that exercise decreased the accumulation of amyloid β-peptide in the hippocampus of AD mice model (Abraham et al., 2019; Hwang et al., 2022). Two other genomics studies confirmed that exercise can delay gene expression alterations and processes associated with hippocampal aging in AD in mice and cause an increase of the majority (>70%) anti-Aging/AD-related gene expression in humans (Alvarez-Lopez et al., 2013; Berchtold et al., 2019). In addition, exercise induces an increase in neurotrophins in AD patients, including the myokine Cathepsin B and BDNF that is closely associated with the status of APOE ε4 in exercisers (Pang et al., 2006; van Dellen et al., 2008; Zajac et al., 2010). Like AD, exercise intervention also plays an active role in Huntington’s disease (HD) and PD. Rear-paw clasping and motor coordination deficit on the static horizontal rod is delayed, with improved striatal deficits, and increased BDNF gene expression in HD mice that have undergone wheel running exercise (Pang et al., 2006; van Dellen et al., 2008; Zajac et al., 2010). Beam walking, rotarod performance, and Quantification of DA neurons have been greatly improved in the PD mice model after undergoing wheel running exercise (Gerecke et al., 2010; Klemann et al., 2018). However, moderate-intensity exercise delayed motor deficit in amyotrophic lateral sclerosis (ALS) mice while high-intensity exercise accelerated it (Carreras et al., 2010). In particular, moderate-intensity like swimming had a better effect on disease remission compared with running (Deforges et al., 2009). Therefore, in addition to universal exercise intervention treatment, we need to consider the influence of different mechanisms of exercise intervention in different diseases and specify differentiated exercise prescriptions.

In addition to the single way of exercise intervention, there are some other imperfections and challenges in current studies. First, most studies are based on animal models. The number and sample size of clinical studies are not large enough which may limit the reliability and consistency of results. Animal and human studies do have some differences, and these differences may be due to the large differences in cell proportions, gene expression levels and hierarchical distribution between human and mouse cerebral cortex, but overall, cell types in the human and mouse cortex are highly conserved. In addition, humans and animals are also different in the means of exercise intervention. Although both of them can be intervened with different intensity and energy supply methods, for quadruped reptiles and upright humans, the activation of muscles, the regulation of nerves and the mobilization of organs are all different. Secondly, most studies are derived from a single omics platform and only a handful of projects are designed with the combination of multiple omics tools. Third, there are lots of confounders that need to be considered. For example, exercise is accompanied by changes in dietary intake. Both metabolome and microbiome are influenced greatly by diet. Studies without the control of diet are unlikely to achieve stable findings. On the other hand, the combined effect of exercise and diet and their interaction may be an interesting but challenging issue worthy of study. Besides diet, gender, age, race, lifestyle, etc. are associated with the alteration of omics features and disease stages. These confounders should get enough attention. Despite these and many other limitations and challenges, identifying omics features and understanding the mechanism of exercise effect on neurodegenerative diseases will have important preventive and therapeutic significance. With the rapid development of omics tools and the widespread recognition of the basic and clinical values of exercise interventions, more efforts will be devoted to this area.

## Author contributions

TC and YG contributed to the formulation of the idea. YG, XC, and SW researched data for the article and wrote the article. YG, TC, SW, XC, DL, YW, and QG made substantial contributions to discussion of content and reviewed/edited the manuscript before submission. All authors contributed to the article and approved the submitted version.

## Funding

This work was funded by the Natural Science Foundation of China (82122012 and 31972935) and National Key R&D Program of China (2019YFA0802300).

## Conflict of interest

The authors declare that the research was conducted in the absence of any commercial or financial relationships that could be construed as a potential conflict of interest.

## Publisher’s note

All claims expressed in this article are solely those of the authors and do not necessarily represent those of their affiliated organizations, or those of the publisher, the editors and the reviewers. Any product that may be evaluated in this article, or claim that may be made by its manufacturer, is not guaranteed or endorsed by the publisher.
